# Glances at the Hospitals

**Published:** 1900-09-29

**Authors:** 


### The Institutional Workshop.

### GLANCES AT THE HOSPITALS.

### THE HARTLEPOOLS HOSPITAL.

It is satisfactory to learn that the managers not only wiped off an adverse balance of £344, but closed the year with a balance in their favour of £401. The total amount of subscriptions was £2,515, and the total expenditure was £2,373. The general subscriptions increased slightly during the year, and so have the church collections and donations; but the marked increase is in the workmen's subscriptions, which jumped from £603 to £1,021. This is a very pleasing point to chronicle. Of the in-patients, 33 were in the hospital at the beginning of the year, and 614 were admitted during the year. The average duration of each patient's residence was only 19 days; the average cost was £3 13s. 4d., and this includes nursing, house repairs, and out-patients' expenses. The average cost per bed was £65 18s. 5d. There were 25 deaths, but 12 of these took place within 24 hours of admission. The number of operations performed was 212, and chloroform was given 194 times. The total number of out-patients was 1,278. We regret to notice that this shows an increase of more than 200 above the number for 1898; but as the workmen's subscriptions have gone up so much, perhaps not much can be said against the additional number of patients. Nevertheless, it is a department we always prefer to see diminishing. A table showing the causes of death is given on page 11. Other tables showing the mortality from the various diseases and the results of the operations would be very desirable.

### ROYAL ALEXANDRA HOSPITAL FOR SICK CHILDREN, BRIGHTON.

The Committee of Management say that, although the hospital has been maintained in an efficient state during the year, they wish to bring under notice the points that the accommodation for out-patients is insufficient, and that more room is required for isolation cases. The total receipts for the general fund were £2,028, and the expenditure was £2,672. There were various legacies received during the year, and the treasurer had a balance of £488 over from the previous year. On January 1st 42 children were in the hospital, and 432 were admitted during the year. Of these, 279 were cured, 95 relieved, 20 discharged for other reasons, 35 died, and 45 remained under treatment. The house surgeon's report consists of five short tables. The first shows the causes of death, but only 34 of the 35 deaths are given. "Tuberculosis meningitis" appears as a cause of death in four cases, but the disease does not appear at all in the list of "diseases from which the in-patients suffered," and "meningitis" is given as the disease in two cases only. The out-patients numbered 2,359 cases, and involved 11,411 attendances. In the orthopædic department there were 23 in-patients and 84 out-patients. The number of attendances was 1,320. All these figures prove that much useful work has been done during the past year, and it should stimulate the inhabitants of the district to increase their annual subscriptions, which at present constitute only one-third of the hospital's income.

### THE CUMBERLAND INFIRMARY.

The total income for the year 1899 was £5,521, but this sum included upwards of £1,300 from legacies and bequests, which, as the committee remark, is a "fluctuating" element, and ought to be placed to a reserve account or treated as extraordinary income. The total ordinary expenditure was £5,035. The average annual cost of maintenance of each patient for provision was £17 8s. 5d., and the average cost per patient per diem was 2s. 11¾d. It is unfortunate that the expenditure was not kept within the income, or that the income was not increased, for we notice that at the end of 1898 there was a balance of £914 due to the bank, and at the end of 1899 this balance had increased to £1,554. A new operating-room was opened during the year, and it cost, including furniture, £1,857. The hospital contained 82 patients on January 1st, and 815 new cases were admitted, making a total of 897 under treatment. Of these 728 were discharged cured or relieved, 38 were incurable, 61 died, and 70 remained in the hospital at the end of the year. The out-patients number 2,050, and these represented 8,598 attendances. The medical and surgical statistics do not seem very clearly arranged. The table on page 11 gives a "list of cases for the year," and we find a total of 4 cases of appendicitis, three of which were cured and 1 relieved. On turning to page 14, which shows the "operations performed during the year," we find a total of 6 cases, of which 4 recovered, 1 died, and 1 was still under treatment. Again, the first table states there were 22 cases of hernia, of which 4 died; and the second table gives 17 cases of hernia operated on, of which 4 died. No doubt these discrepancies could be easily explained by the compiler, but they do not seem clear to the reader.

### THE KIDDERMINSTER INFIRMARY.

The expenditure at this hospital exceeded the income, but only by £25, and the committee point out that the average number of patients has been high, and that £150 has been spent on repairs and improvements. On the first day of 1899 the infirmary contained 43 patients, and 429 were admitted during the year. Of these 284 have been cured, 62 relieved, 29 were made out-patients, 18 were discharged for various other reasons, and 32 died. Of the latter 8 died within forty-eight hours of admission. There were 1,347 out-patients. The average number of beds occupied was 41, and the average duration of each in-patient's stay was 32 days. The medical officers' report gives a list of the diseases from which the patients suffered, and the number of patients afflicted with such diseases, but it tells us nothing about the results of the treatment employed. There is also a statement of the operations performed during the year, but the results of these operations are also absent. We are pleased to find a statement about anæsthetics, and we learn that ether was given 139 times, and chloroform 76. Next year we hope the medical reports will convey more information. This year the operation for the radical cure of hernia was performed 15 times, and yet we are left in the dark as to the number of deaths, if there were any deaths from it.

### THE SOUTH DEVON AND EAST CORNWALL HOSPITAL.

This infirmary is situated at Plymouth. The total receipts from subscriptions, investments, Hospital Sunday and miscellaneous sources amounted to £6,019; and the donations to the general funds amounted to £922. Legacies and special donations and collections exceeded £300. The total expenditure amounted to £6,821, a sum which exceeded the ordinary income by £800. Fortunately, the balance remaining from a bazaar fund of 1898 enabled the debt to be paid, but no reliance can placed on such irregular sources of income; and there still remains a debt to the bankers of £246. Sir Massey Lopes has contributed £4,000 to the funds of the hospital during the year. The hospital contained 92 patients at the beginning of 1899, and 1,152 were admitted during the year. Of these 605 were discharged cured, 48 greatly relieved, 258 relieved, 33 were made out-patients, 85 not improved, 40 for other reasons, 78 died, and 96 remained under treatment. The table showing the operations of the year is very carefully drawn out, and gives all essential details. It would prove an excellent model table for other hospitals. There were 24 cases of ovariotomy or of removal of uterine appendages, and of these 14 were cured, 4 relieved, and 6 died. All the deaths occurred in complicated cases. There were 35 operations for the radical cure of hernia, and all were successful. On page 45 of the report a list of the diseases treated in medical wards is given, but the results are not appended. We notice that anæsthetics were given 785 times, but the kinds used are not specified.

